# Regulation of the *PFK1* gene on the interspecies microbial competition behavior of *Saccharomyces cerevisiae*

**DOI:** 10.1007/s00253-024-13091-9

**Published:** 2024-03-22

**Authors:** Caijuan Zheng, Shuxin Hou, Yu Zhou, Changyuan Yu, Hao Li

**Affiliations:** 1 College of Life Science and Technology, Beijing University of Chemical Technology, Beijing, 100029 People’s Republic of China; 2 School of Public Health, Jining Medical University, Jining, 272067 People’s Republic of China

**Keywords:** *Saccharomyces cerevisiae*, *PFK1*, Interspecies microbial competition, *Escherichia coli*, Ethanol

## Abstract

**Abstract:**

*Saccharomyces cerevisiae* is a widely used strain for ethanol fermentation; meanwhile, efficient utilization of glucose could effectively promote ethanol production. The *PFK1* gene is a key gene for intracellular glucose metabolism in *S. cerevisiae*. Our previous work suggested that although deletion of the *PFK1* gene could confer higher oxidative tolerance to *S. cerevisiae* cells, the *PFK1Δ* strain was prone to contamination by other microorganisms. High interspecies microbial competition ability is vital for the growth and survival of microorganisms in co-cultures. The result of our previous studies hinted us a reasonable logic that the EMP (i.e., the Embden-Meyerhof-Parnas pathway, the glycolytic pathway) key gene *PFK1* could be involved in regulating interspecies competitiveness of *S. cerevisiae* through the regulation of glucose utilization and ethanol production efficiency. The results suggest that under 2% and 5% glucose, the *PFK1Δ* strain showed slower growth than the S288c wild-type and *TDH1Δ* strains in the lag and exponential growth stages, but realized higher growth in the stationary stage. However, relative high supplement of glucose (10%) eliminated this phenomenon, suggesting the importance of glucose in the regulation of *PFK1* in yeast cell growth. Furthermore, during the lag growth phase, the *PFK1Δ* strain displayed a decelerated glucose consumption rate (*P* < 0.05). The expression levels of the *HXT2*, *HXT5*, and *HXT6* genes decreased by approximately 0.5-fold (*P* < 0.05) and the expression level of the *ZWF1* exhibited a onefold increase in the *PFK1Δ* strain compared to that in the *S. cerevisiae* S288c wild-type strain (*P* < 0.05).These findings suggested that the *PFK1* inhibited the uptake and utilization of intracellular glucose by yeast cells, resulting in a higher amount of residual glucose in the medium for the *PFK1Δ* strain to utilize for growth during the reverse overshoot stage in the stationary phase. The results presented here also indicated the potential of ethanol as a defensive weapon against *S. cerevisiae*. The lower ethanol yield in the early stage of the *PFK1Δ* strain (*P* < 0.001) and the decreased expression levels of the *PDC5* and *PDC6* (*P* < 0.05), which led to slower growth, resulted in the strain being less competitive than the wild-type strain when co-cultured with *Escherichia coli*. The lower interspecies competitiveness of the *PFK1Δ* strain further promoted the growth of co-cultured *E. coli*, which in turn activated the ethanol production efficiency of the *PFK1Δ* strain to antagonize it from *E. coli* at the stationary stage. The results presented clarified the regulation of the *PFK1* gene on the growth and interspecies microbial competition behavior of *S. cerevisiae* and would help us to understand the microbial interactions between *S. cerevisiae* and other microorganisms.

**Key points:**

• *PFK1Δ strain could realize reverse growth overshoot at the stationary stage*

• *PFK1 deletion decreased ethanol yield and interspecific competitiveness*

• *Proportion of E. coli in co-culture affected ethanol yield capacity of yeast cells*

**Supplementary Information:**

The online version contains supplementary material available at 10.1007/s00253-024-13091-9.

## Introduction

*Saccharomyces cerevisiae* is a commonly used species for industrial fermentation (Madhavan et al. 2021), such as the production of beverages, bioethanol, and other chemicals (Lacerda et al. 2020). It is often used in co-fermentation with other microorganisms to promote fermentation efficiency. For example, to add a unique flavor, fermentation by *S. cerevisiae* is usually co-cultured with other microorganisms (Yılmaz and Gökmen 2021; Soden et al. 2008), such as non-*Saccharomyces* yeast and lactic acid bacteria (Sieuwerts et al. 2018; Jin et al. 2021); meanwhile, and to improve the production efficiency, *Acremonium cellulolyticus* C-1 was co-cultured with *S. cerevisiae* for realizing the simultaneous saccharification and fermentation of ethanol by a one-pot method without any pretreatment or addition of cellulase (Park et al. 2012).

In mixed fermentation, a microsystem is formed between different microorganisms (Pandhal and Noirel 2014). There are many complex interactions between microorganisms in microsystems like these, including competition, mutualism, amensalism, and predation (Zengler and Zaramela 2018). For example, in the sesame wine fermentation process, although there is a competitive relationship among the five dominant microorganisms (i.e., *S. cerevisiae*, *Bacillus licheniformis*, *Pichia membranaefaciens*, *Bacillus amyloliquefaciens*, and *Issatchenkia orientalis*), their cooperation also facilitates the production of most flavor compounds (Wu et al. 2014). Similarly, our previous research also indicated that co-cultivated *Lactobacillus plantarum* not only inhibits the growth of *S. cerevisiae* (Dong et al. 2015), but also enhances the oxidative and ethanol tolerance of yeast cells (He et al. 2021; Kang et al. 2021).

Microbial metabolic activity may modulate the cellular behavior such as tolerance (Kotte et al. 2010; Heinemann and Sauer 2010). Meanwhile, co-cultured *L. plantarum* associated with regulation of carbohydrate metabolism (Dong et al. 2015) and higher oxidative and ethanol tolerance of *S. cerevisiae* also hinted at a possible correlation between central carbon metabolism and tolerance in yeast cells. To test this hypothesis, five different mutant strains with deletion of the EMP (i.e., the Embden-Meyerhof-Parnas pathway, the glycolytic pathway) key genes *HXK1*, *ENO1*, *TDH1*, *PFK1*, and *FBP1* were constructed from *S. cerevisiae* S288c strain using the CRISPR-Cas9 strategy, and the results suggested that the *PFK1Δ* strain always showed slower growth in the early growth stage but reversed overshoot in the later stage in comparison to the wild strain and the other four mutant strains (Kang 2022). Interestingly, under the same culture conditions, the *PFK1Δ* strain was more susceptible to invasion by other microorganisms (i.e., *Escherichia coli* and *Acinetobacter nosocomialis*) compared to that of the wild-type strain and the other four mutant strains. These results preliminarily suggest the *PFK1* gene regulates on the interspecies competition behavior of *S. cerevisiae*.

Microorganisms have evolved various weapons, both defensive and offensive, in their evolutionary process to enhance their survival capabilities and maximize their own benefits (Granato et al. 2019). For instance, bacteria in the soil may compete for scarce resources by either manufacturing toxins or enhancing their motility (Hibbing et al. 2010; Ghoul and Mitri 2016). Streptomycin produced by the filamentous bacterium *Streptomyces griseus* can be used as a defensive and offensive weapon to kill some susceptible species to prevent their invasion, thereby improving the competitiveness of *S. griseus* (Westhoff et al. 2020). Similar to the “The Red Queen hypothesis” (Strotz et al. 2018), interspecific relationships can also promote the evolution of species under certain environmental conditions. In a similar vein, *S. cerevisiae* employs strategies to enhance its competitiveness, either proactively or passively, while it coexists with other microorganisms. When the benefits of such strategies are greater than the input, it will continue to evolve more favorable strains in this direction. Meanwhile, ethanol has been effectively used in disinfection and sterilization in many fields (Mathew and Goyal 2023). Ethanol, as a metabolite of *S. cerevisiae*, exerted inhibitory effects on microorganism growth at high concentrations. Elevated ethanol concentrations could disrupt cell membrane integrity and functionality, thereby interfering with intracellular metabolism and compromising cell physiological activities (Patra et al. 2006). It is worth noting that different microorganisms may possess varying degrees of ethanol tolerance (Ingram 1990), which could influence the competitive dynamics between *S. cerevisiae* and other microorganisms. In nature, *S. cerevisiae* can tolerate higher ethanol concentrations than other microorganisms (Pina et al. 2004; Arroyo-López et al. 2010) to enable its survival and proliferation even in environments with relatively high ethanol concentrations, suggesting that ethanol might serve as a defense weapon for yeast to improve its viability and antagonize other microorganisms to maximize its benefits during co-existence. As a result, it can be logically inferred that a greater ethanol concentration will lead to improved competitiveness and survivability of *S. cerevisiae* when coexisting with other microorganisms, whereas a lower ethanol concentration will be less favorable for its survival. Based on our previous findings, we can propose a plausible hypothesis that the *PFK1* gene plays a crucial role in regulating the ethanol production ability of *S. cerevisiae* by controlling its glucose uptake and utilization, which ultimately affects the interspecies competitiveness of *S. cerevisiae*.

Here, the growth under different concentrations of glucose supplements, glucose metabolism, and ethanol production of three strains (i.e., S288c wild-type, *PFK1Δ*, and *TDH1Δ* strains) were measured, and the expression of related metabolic genes between the S288c wild-type and *PFK1Δ* strains was compared. Moreover, the *E. coli* co-culture-associated biomass change and the expression of genes involved in ethanol synthesis of the S288c wild-type and *PFK1Δ* strain were also determined. The results presented here will help us understand the regulation of the *PFK1* gene on the growth and interspecies microbial competition behavior of *S. cerevisiae*.

## Materials and methods

### Strains, media, and cultural conditions

*S. cerevisiae* S288c strain (ATCC 204508) was purchased from the Institute of Microbiology of the Chinese Academy of Sciences (IMCAS). The *PFK1Δ* and *TDH1Δ* strains were constructions from the S288c strain using the CRISPR-Cas9 strategy. *S. cerevisiae* S288c wild-type, *PFK1Δ*, and *TDH1Δ* strains were cultured in 250-ml conical flasks containing 100-ml YPD broth (2% glucose, 2% peptone, and 1% yeast extract) at 30 °C and shaken at 150 rpm. *E. coli* BL21 (DE3) was also purchased from Institute of Microbiology of the Chinese Academy of Sciences (IMCAS), and cultured in 250-ml conical flasks containing 100 ml of LB broth (1% peptone, 0.5% yeast extract, 1% sodium chloride) at 37 °C and shaken at 180 rpm.

### Determination of growth differences of three yeast strains supplied with various concentrations of glucose

The S288c, *PFK1Δ*, and *TDH1Δ* strains were inoculated into 100-ml YPD medium with an inoculation cell density of 4 × 10^5^ cfu/ml (i.e., colony forming units per milliliter), and the OD_600_ value was measured by using an UV spectrophotometer after sampling every 2 h to determine the growth curve. The concentration of glucose in the medium was increased to 5% and 10%, respectively, to observe the impact of different carbon sources on microbial metabolism and growth. Samples were taken every 4 h to measure the OD_600_ value. The growth rate was calculated using the following formula:

$$\mu =\frac{{\text{ln}} {X}_{2}-{\text{ln}} {X}_{1}}{{t}_{2}-{t}_{1}}$$

*X*_2_ and *X*_1_ refer to the OD_600_ values of cell cultures at culture time (*t*) *t*_1_ and *t*_2_, respectively.

### Determination of the contents of extracellular and intracellular glucose

The contents of extracellular and intracellular glucose in yeast during cultivation were determined by HPLC (high-performance liquid chromatography; Shimadzu, i-Series, Kyoto, Japan) analysis. The chromatographic column was Bio-Rad HPX-87H (9 μm, 300 × 7.8 mm, CA, USA), the mobile phase was 7 mmol/l H_2_SO_4_, the column temperature was 55 °C, and the flow rate was 0.6 ml/min. To determine the extracellular glucose content, the initial inoculation densities of the three strains were all 4 × 10^5^ cfu/ml, and 1.5 ml of cultured suspension was collected from 12 h, 14 h, 16 h, and 20 h of cultivation. After centrifugation, the supernatant was added into a liquid injection vial through a 0.22-μm filter for HPLC analysis. To measure the intracellular glucose levels, a 50-ml culture sample was collected from the yeast cells after 20 h of cultivation. The supernatant was then removed by centrifugation, and the cells were washed 3 times with 1 × PBS before being resuspended in 25 ml of deionized water. Repeated freeze–thaw cycles and ultrasonic crushing (ultrasonic power 400 W, radiation time 11 s, total time 14.5 min) were performed to break the yeast cell wall. After centrifugation, the supernatant was filtered through a 0.22-μm filter for HPLC analysis.

Moreover, the extracellular ethanol contents at the 12, 14, 16, and 20 h of cultivation were also determined by HPLC analysis.

### Determination of expressions of genes involved in the hexose transporter family, pentose phosphate pathway, and ethanol synthesis

Key genes involved in the hexose transporter family, pentose phosphate pathway, and ethanol synthesis were chosen to determine the *PFK1* deletion-associated expression level changes. Yeast cells were collected after 10 and 20 h of cultivation. Primers used in this work were designed using Primer Premier 5 (PREMIER Biosoft International, San Francisco, CA, USA) and are listed in Table 1. RNA extraction, reverse transcription, and qRT-PCR analysis were performed following the procedure reported in our previous work (Dong et al. 2015).

**Table 1:** Oligonucleotides used in this study

Primers	Sequences (5′ to 3′)
ACT1-F/R	TGCCCCAGAAGAACACCCTG/CAAAACGGCTTGGATGGAAAC
HXT2-F/R	GTTTGATGTGCGTCGTTCTGG/GCAATACCACCGACACCCATA
HXT5-F/R	GTCGTCTATGCCTCTGTTGGT/GCCCAAGTGGTAGCGAAAC
HXT6-F/R	GGTTGTGGGGTTTCTTGATTGG/TCTTCCCACATGGTGTTGACT
HXT7-F/R	CGTTATTTGGCTGAAGTCGGT/CCTCTACACCAGCCAAGACAG
ZWF1-F/R	CCAGAGGCTTACGAGGTGTT/GGACGCTCTATGTGCTTCAGT
ADH1-F/R	GTCGGTGCTGTTCTAAAGGC/GCTGGCATACCGACCAAAAC
PDC1-F/R	CCGCTAAGGGTTACAAGCCA/GGAGGTACCGGTTTCAGCAA
PDC5-F/R	CCAAACGACGCTGAAGCTGA/CTAGAAGCACAAGCATCAGCCA
PDC6-F/R	CCAACCAAAACTCCCGCAAA/CCCACAACACCTGCGAGATA

### Evaluation of the potential ability of ethanol to antagonize *E. coli*

To test whether ethanol could be used as a defensive weapon for yeast cells, physically quarantined *S. cerevisiae* secreted ethanol content in the presence or absence of *E. coli* was determined (Supplemental Fig. S1A). A physical quarantine system was constructed using three minimum inhibitory concentration (MIC) bottles that were separated from each other with a 0.45-μm filter membrane. In the physical quarantine system, microorganisms cultured in different bottles cannot touch each other, while the secreted molecules (i.e., ethanol) can freely permeate the filter membrane and enter the other bottles. *S. cerevisiae* (4 × 10^5^ cfu/ml) was inoculated into the middle bottle, and *E. coli* (1 × 10^8^ cfu/ml) was inoculated into the left bottle, while the right side bottle without any inoculation was considered as the blank control. The ethanol content in each bottle was determined through HPLC analysis after 10 or 20 h of cultivation. Meanwhile, to further eliminate the effects of *S. cerevisiae* cell wall and other substances on *E. coli*, the yeast cell wall was degraded and the supernatant was replaced. Approximately 5 × 10^7^ cells from *S. cerevisiae* suspension cultured in the exponential phase were centrifuged and treated with yeast wall-breaking enzyme solution (Solarbio, Beijing, China) at 30 °C for 2 h. Protoplasts were inoculated into an LB medium containing *E. coli* and cultured for 1 h. The number of *E. coli* was calculated using an LB agar medium containing 10 mg/l natamycin (Liu et al. 2017). Normal *S. cerevisiae* cells were used as the control. In the other group, *E. coli* culture was suspended and centrifuged to separate the precipitate. This precipitate was then added to the supernatant of *S. cerevisiae* and the YPD liquid medium containing a certain concentration of ethanol separately (i.e., ethanol produced by *S. cerevisiae* cultured for 10 h). *E. coli* treated with YPD was used as a control. The growth of *E. coli* was compared to the LB agar medium counting method after culturing at 37 °C for 1 h.

Finally, according to the ethanol concentration produced by *S. cerevisiae* at 10, 14, 16, and 20 h, different concentrations of ethanol solution were prepared and added to *E. coli* cultured to the exponential phase for 1 h. The agar plate method was used to count the diluted bacterial suspension, and the effects of varying ethanol concentrations on the growth of *E. coli* were compared.

### Determination of the growth of *S. cerevisiae* and *E. coli* in the co-culture

To evaluate the susceptibility of the *PFK1Δ* strain to other microorganisms (i.e., *E. coli* in this study), one type of *S. cerevisiae* strain (i.e., S288c wild-type, *PFK1Δ* type, or *TDH1Δ* type) was co-inoculated with *E. coli* into the YPD medium, and the growth of yeast and *E. coli* were, respectively, determined every 4 h. To simulate the co-culture system and ensure that the growth was easy to determine, the inoculation cell densities of *S. cerevisiae* and *E. coli* were 4 × 10^5^ cfu/ml and 1 × 10^6^ cfu/ml, respectively. The number of *S. cerevisiae* cells was determined by microscopy, and the biomass of *E. coli* was measured by counting the number of cfu on LB agar medium containing 10 mg/l natamycin after 24 h of culture (Liu et al. 2017).

Moreover, to evaluate the *E. coli* co-culture associated with ethanol production difference between the two yeast strains (i.e., S288c wild-type and *PFK1Δ* type), key genes involved in ethanol synthesis were chosen to determine their expression level changes. To ensure the accuracy of the expression level analysis of yeast genes, we removed *E. coli* cells from the co-culture system using the G5 (2–4-μm pore diameter) sand core funnel, following our previous method (Dong et al. 2015). Following the collection of yeast cells from the funnel, RNA was obtained for reverse transcription, and gene expression was evaluated through quantitative reverse transcription polymerase chain reaction (qRT-PCR).

### Statistical analysis

An independent-sample *T* test was performed on specific metabolites and genes using SPSS software (version 20.0; IBM Corp., Armonk, NY USA), to assess the statistical significance of the metabolic changes, and the error bars correspond to standard error. All experiments were run in triplicate to quintuplicate. Differences showing *P*-values less than 0.05 were considered statistically.

## Results

### Growth differences among *S. cerevisiae *S288c wild-type, *PFK1Δ*, and *TDH1Δ* strains

**Fig. 1:**
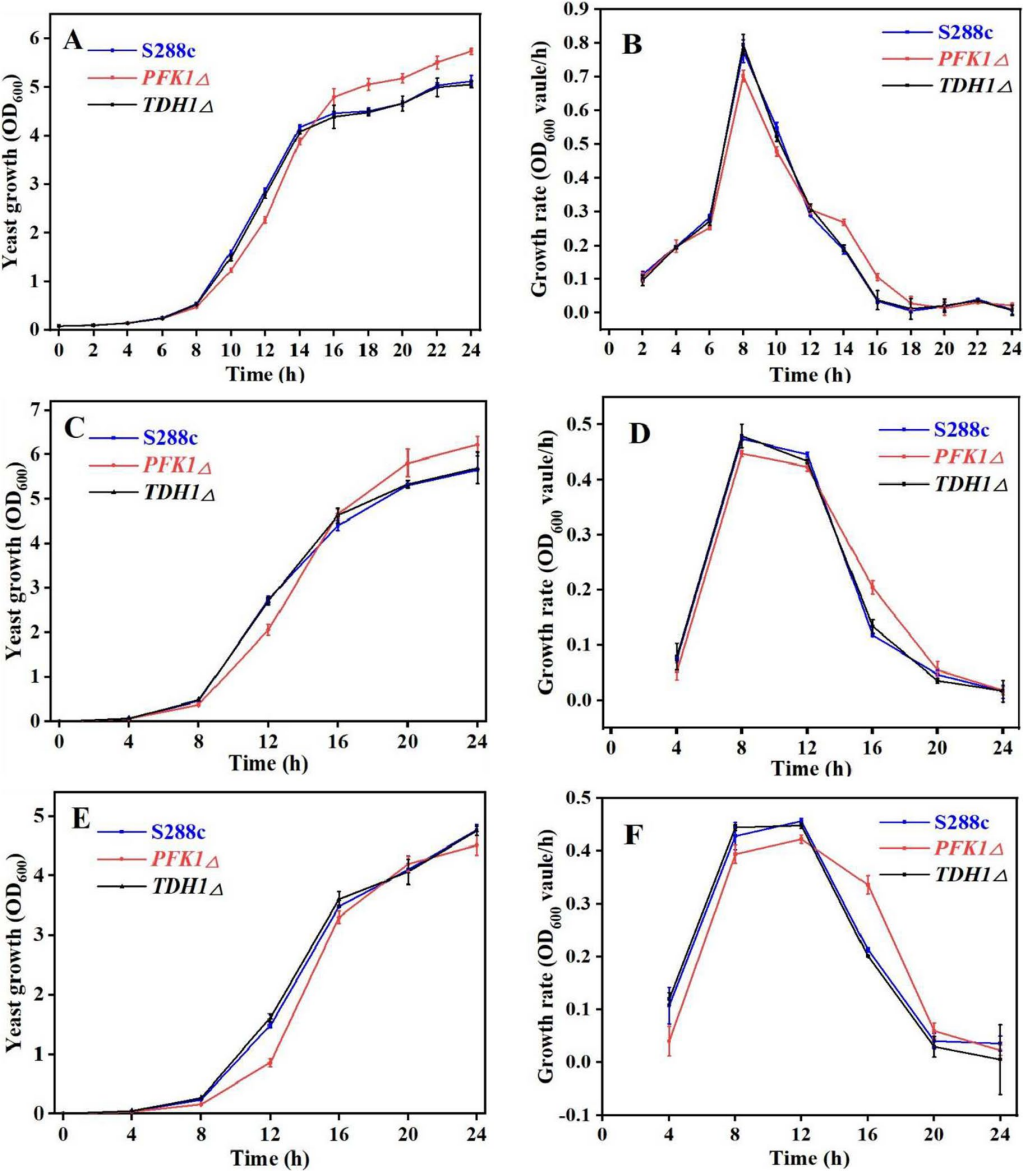
Growth curve of the *Saccharomyces cerevisiae* S288c wild-type, *PFK1Δ*, and *TDH1Δ* strains under different sugar concentrations. **A** The growth curve of the three strains was determined every 2 h under a 2% glucose concentration in the culture medium, intending to identify the range of inflection points. **B** The growth rate of the three strains under a 2% glucose concentration. **C** Under the condition of 5% glucose concentration in the culture medium, to determine whether there was still an anti-overshoot phenomenon, the growth curves of three strains were obtained by measuring the OD_600_ value every 4 h. **D** The growth rate of the three strains under a 5% glucose concentration. **E** Growth curves of three strains under the condition of medium containing 10% glucose concentration. OD_600_ values were measured every 4 h. **F** The growth rate of the three strains under a 10% glucose concentration

The *PFK1Δ* strain showed slower growth than the S288c wild-type and the *TDH1Δ* strain in the lag and exponential growth stages but showed higher growth in the stationary stage at of 2% glucose (Fig. 1 A and B). The growth of the *TDH1Δ* mutant strain was not noticeably different from that of the wild-type strain (Fig. 1A). The three strains showed similar growth patterns when the glucose concentration in the medium was increased to 5%, as it was under 2% glucose concentration (Fig. 1C). When the glucose concentration in the medium was further increased to 10%, although lower growth biomass of the *PFK1Δ* strain in the lag and exponential stages still existed, its higher growth biomass in the stationary stage was not detected (Fig. 1E). Consequently, varying concentrations of extracellular and intracellular glucose can influence the proliferation of the three microbial strains. The supply of carbon sources in the later stages of growth is essential to realize growth reversal.

### Differences in intracellular and extracellular glucose contents, and ethanol yield among *S. cerevisiae *S288c wild-type, *PFK1Δ*, and *TDH1Δ* strains

**Fig. 2:**
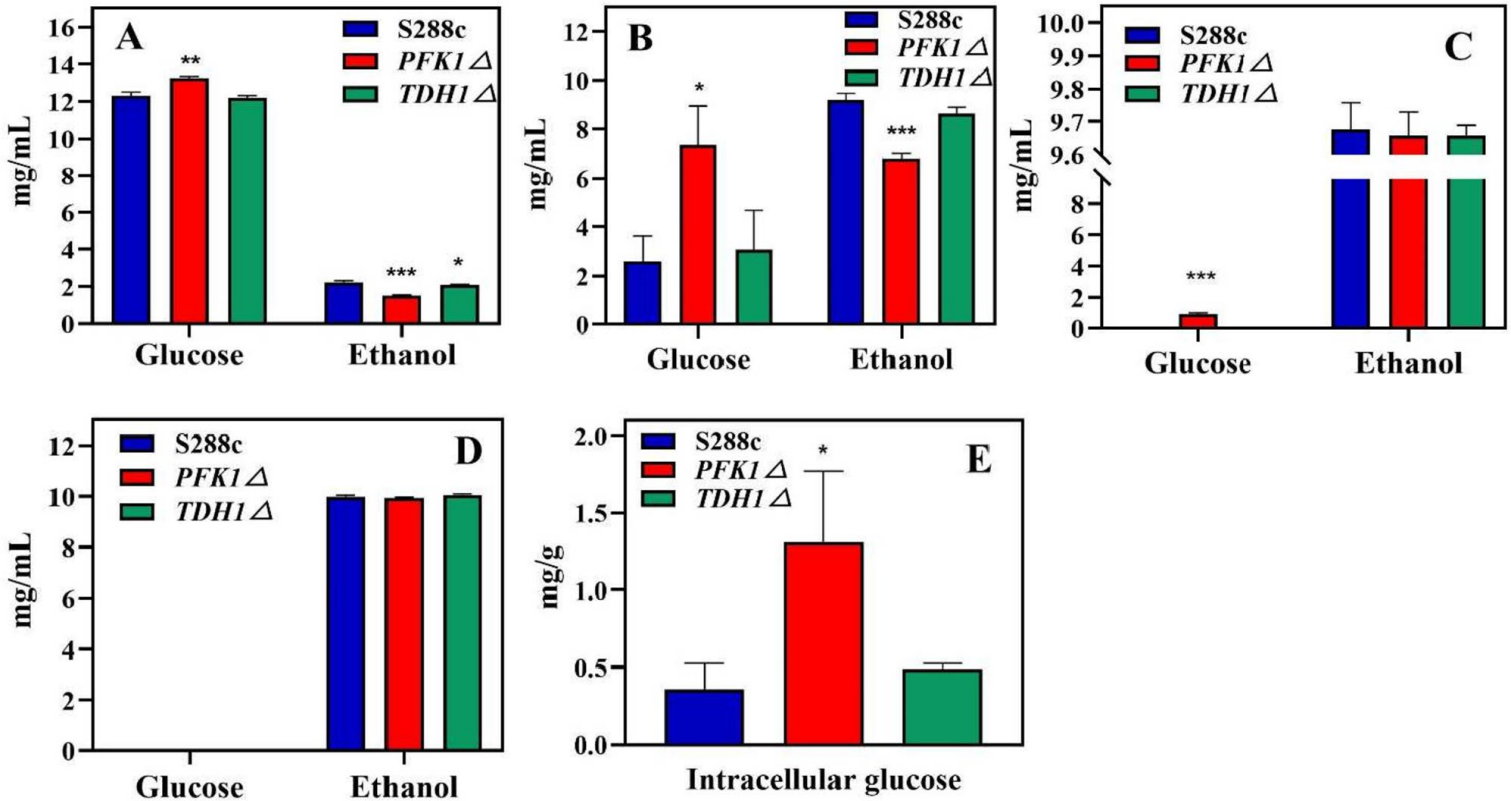
Comparison of sugar consumption and ethanol yield of three strains at different time points. The concentration of extracellular glucose and ethanol of each of the three strains was measured at 10 h of incubation (**A**), at 14 h of incubation (**B**), at 16 h of incubation (**C**), and at 20 h of incubation (**D**). **E** Intracellular glucose content of three strains after the cell wall broken at 20 h. *P < 0.05 compared to the S288c wild-type strain. **P < 0.01 compared to the S288c wild-type strain. ***P < 0.001 compared to the wild-type S288c strain

Two time points were chosen — one before and one after a critical point — in order to monitor glucose utilization and ethanol production, and to validate the impact of varying glucose levels on cell growth. In the exponential phase (i.e., 10 and 14 h of cultivation), the extracellular glucose level of *PFK1Δ* (7.33 mg/ml) strain was higher than that of the S288c (2.60 mg/ml) and *TDH1Δ* (3.08 mg/ml) strains (*P* < 0.05) (Fig. 2 A and B). In the early stationary phase (i.e., 16 h of cultivation), only the *PFK1Δ* strain retained extracellular glucose with a concentration of 0.9 mg/ml (Fig. 2C). The yield of ethanol produced by the *PFK1Δ* strain was lower than that produced by S288c and *TDH1Δ* strains (*P* < 0.05) (Fig. 2 A and B). However, the ethanol fermentation concentration of the three strains could reach 10 mg/ml at 20 h (Fig. 2D). Meanwhile, the glucose content of the *PFK1Δ* strain (1.3 mg/g) was higher than that of the S288c (0.36 mg/g) and *TDH1Δ* strains (0.49 mg/g) after 20 h of cultivation (*P* < 0.05) (Fig. 2E).

### Effect of *PFK1* gene deletion on hexose transporter family, pentose phosphate pathway, and ethanol synthesis gene expression in *S. cerevisiae*

**Fig. 3:**
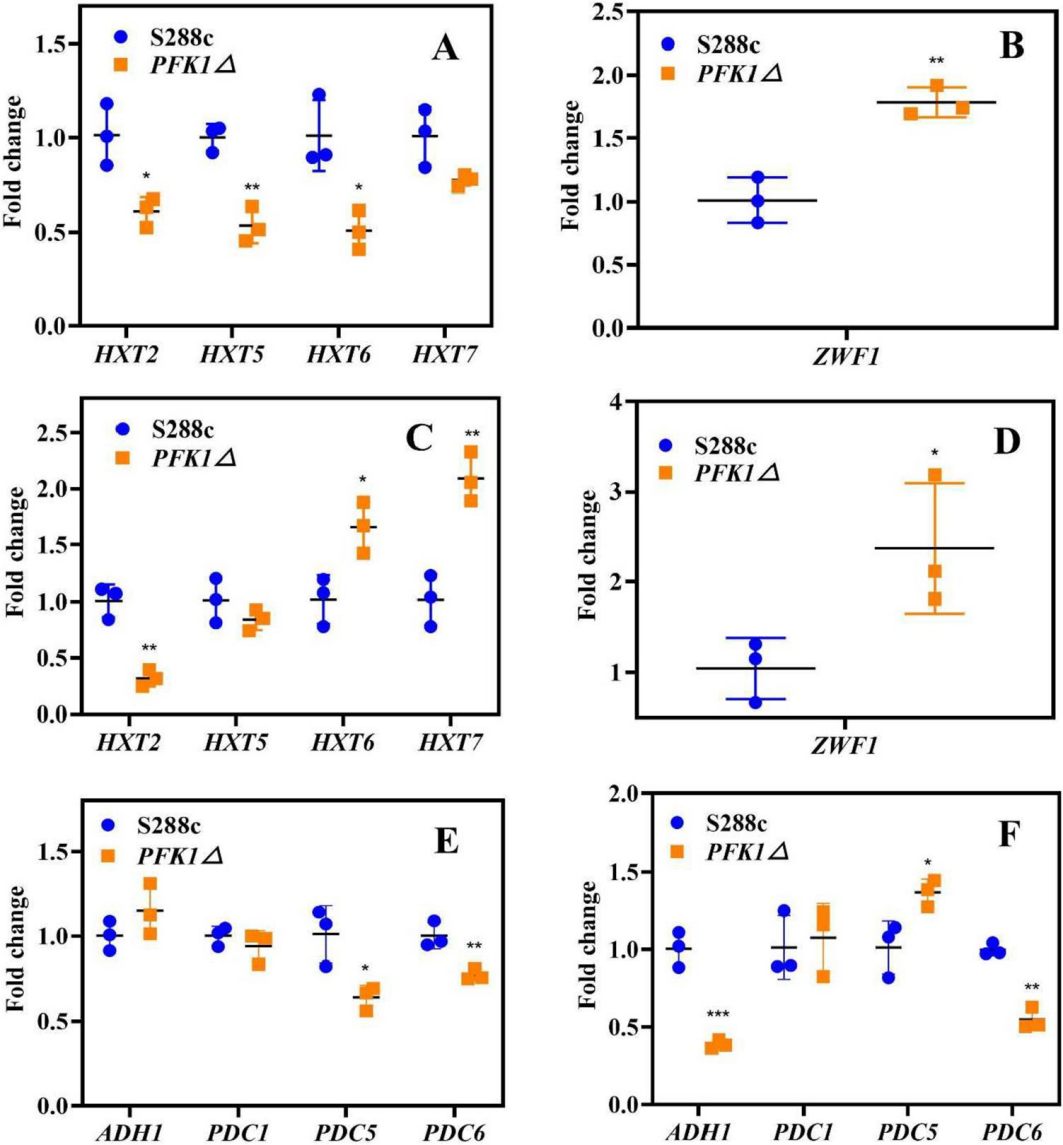
Effect of *PFK1* gene deletion on hexose transporter family, pentose phosphate pathway, and ethanol synthesis gene expression in *S. cerevisiae*. Comparison of expression levels of key genes *HXT2*, *HXT5*, *HXT6*, and *HXT7* controlling glucose transport in the S288c wild-type strain and the *PFK1Δ* strain at 10 h (**A**) and 20 h (**C**). Comparison of expression levels of *ZWF1*, a key gene controlling HMP pathway, in S288c wild-type and *PFK1Δ* strains at 10 h (**B**) and 20 h (**D**). Comparison of expression levels of key genes *ADH1*, *PDC1*, *PDC5*, and *PDC6* controlling ethanol synthesis in the S288c wild-type and the *PFK1Δ* strains at 8 h (**E**) and 16 h (**F**). *P < 0.05 compared to the S288c wild-type strain. **P < 0.01 compared to the S288c wild-type strain

*HXT2*, *HXT5*, and *HXT6/7*, which are the key genes involved in glucose transport in *S. cerevisiae*, suggest low, intermediate, and high affinity for glucose, respectively (Buziol et al. 2008; Ye et al. 2001). After 10 h of cultivation, the *PFK1Δ* strain exhibited a 0.5-fold decrease in the expression levels of *HXT2*, *HXT5*, and *HXT6* compared to the *S. cerevisiae* S288c wild-type strain (*P* < 0.05) (Fig. 3A). However, the expression of *ZWF1*, the key gene in the pentose phosphate pathway (HMP) (DangThu et al. 2020), increased by 0.8-fold in *PFK1Δ* strain after 10 h of cultivation (*P* < 0.05) (Fig. 3B). Although the expression of *HXT2* and *HXT5* did not change or even decreased at 20 h of cultivation, the expression levels of *HXT6* and *HXT7*, which have a high affinity for glucose, exhibited 0.6-fold and onefold increases, respectively. (*P* < 0.05) (Fig. 3C). At this time, the expression level of *ZWF1* in *PFK1Δ* strain still exhibited a onefold increase (*P* < 0.05) (Fig. 3D).

Pyruvate decarboxylase ( encoded by *PDC*) and alcohol dehydrogenase (from gene *ADH*) were the key enzymes involved in ethanol synthesis (Strommer and Garabagi 2008). However, *ADH* is more critical than *PDC* for ethanol biosynthesis (Ke et al. 2015). Here, the *PFK1* gene deletion was associated with lower expressions of *PDC5* and *PDC6* (*P* < 0.05) (Fig. 3E) in the early stage of growth. During the stationary growth stage, although the expression of the *PDC5* gene was upregulated in *PFK1Δ* strain (*P* < 0.05) (Fig. 3F), the down-regulation of the *ADH1* gene (*P* < 0.001) limited the ethanol production efficiency of *PFK1Δ* strain (Fig. 3F).

### Antagonistic effect of ethanol on *E. coli*

After co-cultivation with intact yeast cells and *S. cerevisiae* protoplasts, the *E. coli* biomass of both groups was about 1.7 × 10^9^ cfu/ml, which was significantly lower than that of 2.23 × 10^9^ cfu/ml of the control group (*P* < 0.01) (Supplemental Fig. S1B). Through the physical isolation of MIC bottles, the ethanol concentration of the experimental group inoculated with *E. coli* was 0.292 mg/ml at 10 h, which was significantly higher than that of the uninoculated blank group (0. 04 mg/ml) (*P* < 0.001) (Supplemental Fig. S2). Simultaneously, the concentration of ethanol in separately cultured *E. coli* (0.133 mg/ml) was determined to eliminate self-interference. After using the supernatant of cultured *S. cerevisiae* and the YPD liquid medium containing 2.23 mg/ml ethanol (i.e., the ethanol concentration in the supernatant of *S. cerevisiae* cultured for 10 h) to replace the supernatant of *E. coli* for 1 h, the biomass of *E. coli* decreased by about 13%, respectively (*P* < 0.05) (Supplemental Fig. S3). Based on the amount of ethanol produced by *S. cerevisiae* at four different time points, the corresponding concentration of ethanol solution was prepared to treat *E. coli*, and the change in biomass with ethanol concentration was determined. *E. coli* biomass decreased after ethanol treatment at different concentrations (Supplemental Fig. S4), indicating that ethanol had an obvious inhibitory effect on growth of *E. coli*.

### Differences of growth of *S. cerevisiae *S288c wild-type, *PFK1Δ*, and *TDH1Δ* strains in co-culture with *E. coli* and expressions of key genes involved in ethanol synthesis

**Fig. 4:**
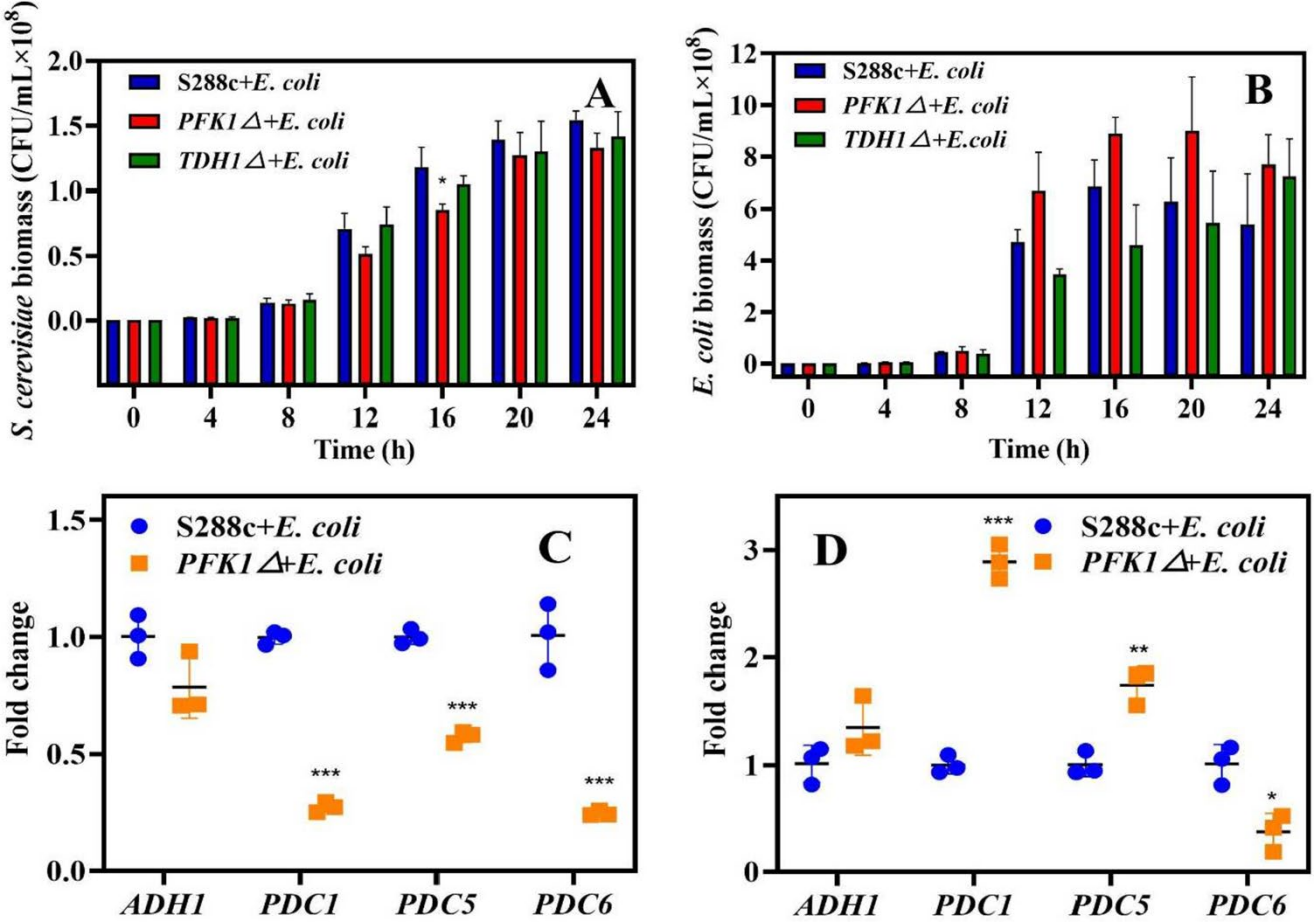
Growth of the *Saccharomyces cerevisiae* S288c wild-type, *PFK1Δ*, and *TDH1Δ* strains, and *Escherichia coli* co-cultured, respectively, and expression of key genes in ethanol synthesis after co-culture. **A** Biomass of the *E. coli* at different times after co-culture. **B** Biomass of the S288c wild-type, *PFK1Δ*, and *TDH1Δ* strains, at different times after co-culture. Differences in gene expression of *ADH1*, *PDC1*, *PDC5*, and *PDC6* in co-culture of two strains and *Escherichia coli* at 8 h (**C**) and 16 h (**D**). *P < 0.05 compared to the S288c wild-type strain. **P < 0.01 compared to the S288c wild-type strain. ***P < 0.001 compared to the S288c wild-type strain

Growth of the *PFK1Δ* strain in the mixed culture was slower than that of *S. cerevisiae* S288c wild-type and *TDH1Δ* strains, and the *PFK1Δ* strain did not achieve higher biomass growth at the stationary stage (Fig. 4A). Meanwhile, the biomass of *E. coli* in co-culture with the *PFK1Δ* strain was the highest compared to that in co-culture with the other two strains (i.e., *S. cerevisiae* S288c wild-type and *TDH1Δ* strains) (Fig. 4B).

Based on the increase in mixed culture, 8 and 16 h were chosen as the time points for evaluating the expression of genes that play a crucial role in ethanol synthesis. After 8 h of mixed culture, the expression levels of *PDC1*, *PDC5*, and *PDC6* in the *PFK1Δ* strain decreased by approximately 0.5-fold compared to the wild-type strain (*P* < 0.001) (Fig. 4C). But after 16 h of mixed culture, the expressions of *ADH1*, *PDC1*, and *PDC5* in the *PFK1Δ* strain showed various degrees of upregulation (*P* < 0.01) (Fig. 4D). This showed that the presence of *E. coli* had a certain effect on ethanol synthesis in the *PFK1Δ* strain.

## Discussion

*S. cerevisiae*, a commonly used model organism in industry, is prone to contamination by other microorganisms during fermentation (Nevoigt 2008), and interspecific competitiveness is crucial for the growth and survival of microorganisms (Hibbing et al. 2010). Out of the five strains of *S. cerevisiae* that had key genes involved in glucose metabolism knocked out to enhance its oxidation tolerance, the *PFK1Δ* strain displayed growth lag and reverse overshoot (Fig. 1A), and was frequently contaminated by other microorganisms. Previous studies have suggested that the utilization of glucose by *PFK1* is critical (Yuan et al. 2020; Arvanitidis and Heinisch 1994), whereas the *TDH*1 gene is less critical for the use of glucose (Randez-Gil et al. 2020). The microbial growth can also be affected by nutrients, especially carbon sources (Olivares-Marin et al. 2018); there was no growth biomass anti-overshoot under the condition of a sufficient carbon source (Fig. 1E). Based on this, the lower growth of *PFK1Δ* in the lag and exponential growth stages (as shown in Fig. 1) indicates that the strain utilizes less glucose than the other two strains, which results in more residual glucose being available for *PFK1Δ* to use in the stationary phase, leading to higher growth. The data presented in Fig. 2 indicates that the glucose uptake and utilization efficiency of the *PFK1Δ* strain is lower than that of the other two strains, with respect to extracellular glucose residue content at different time points. The amount of residual sugar in the culture system could affect the growth of *S. cerevisiae* (Jansen et al. 2005; Nissen et al. 2003), so that more residual glucose remaining in the lag and exponential stages also provided the material base for the subsequent growth surpassing of the *PFK1Δ* strain in the stationary phase. Intracellular glucose at 20 h also confirmed the higher growth of the *PFK1Δ* strain than the other two strains in the stationary stage (Fig. 2E). Previous studies have demonstrated that the activity of the *PFK1* gene can influence glycolysis flux, and this can subsequently impact the efficiency of glucose transport in cells (Papagianni and Avramidis 2011). The lower expression of *HXT2*, *HXT5*, and *HXT6* in the *PFK1Δ* strain compared to that in the *S. cerevisiae* S288c wild-type strain (*P* < 0.05) (Fig. 3A) resulted in a lower glucose transport efficiency of in the *PFK1Δ* strain. Therefore, there was a greater extracellular glucose surplus in the *PFK1Δ* strain during the early phase (Fig. 2). Deletion of the key gene *PFK1* in the glycolysis pathway (i.e., EMP, the Embden-Meyerhof-Parnas pathway) decreased the glucose utilization efficiency of *S. cerevisiae*, which in turn further affected its transport. Deletion of the key gene, *PFK1* combined with decreased glucose transport efficiency, contributed to the lower growth rate of the *PFK1Δ* strain in the lag and exponential stages. However, on the other hand, microbes would survive as much as possible by adopting different strategies in extreme cases (Dumorné et al. 2017). Although the pentose phosphate pathway (HMP) was not the primary pathway for *S. cerevisiae* to consume carbon sources, such as glucose, when the Embden-Meyerhof-Parnas pathway was inhibited or blocked, HMP was activated as much as possible to ensure yeast survival (Li et al. 2012). Therefore, activation of the HMP pathway in the *PFK1Δ* strain was shown by an increase of *ZWF1* gene expression (Fig. 3 B and D). The *PFK1Δ* strain consumed more residual glucose during the stationary phase at 20 h of culture compared to the forward growth phase, indicating a potential for reverse overshoot. The increased expression of *HXT6* and *HXT7* with high affinity for glucose (Fig. 3C) also indicated that the *PFK1Δ* strain continued to utilize glucose for growth in the later growth stage.

Ethanol is the main fermentation product of *S. cerevisiae* and glucose metabolism often affects ethanol synthesis (Sabater-Muñoz et al. 2020; Yang et al. 2022). The production of ethanol by the *PFK1Δ* strain was lower than that of the wild-type S288c and *TDH1Δ* strains of *S. cerevisiae* (as shown in Fig. 2 A and B), indicating poor growth during the exponential phase. Pyruvate is catalyzed by pyruvate decarboxylase (i.e., PDC) to produce acetaldehyde, which is further reduced by alcohol dehydrogenase (i.e., ADH) to form ethanol (Mithran et al. 2014). Although the expression level of *PDC1* in *S. cerevisiae* is higher than that of *PDC5* and *PDC6* (Raj et al. 2014; Hohmann 1991), the catalytic efficiency of *PDC5* is highest when the pyruvate amount is sufficient (Agarwal et al. 2013). In the early growth phase, the gene expression of *PDC5* and *PDC6* in the *PFK1Δ* strain decreased (*P* < 0.05) (Fig. 3E), indicating lower ethanol production efficiency in the *PFK1Δ* strain in the early growth stage. This result was also confirmed by the lower ethanol yield of the *PFK1Δ* strain during the early growth stage (Fig. 2 A and B). As the stationary phase was reached, despite an increase in *PDC5* gene expression (Fig. 3F), the more crucial *ADH1* gene expression decreased, hindering the ethanol synthesis efficiency in the *PFK1Δ* strain. On the other hand, the higher biomass of *PFK1Δ* at the stationary stage (Fig. 1A) was responsible for the similar ethanol yield of these two strains (Fig. 2D). The deletion of the *PFK1* gene hindered the EMP pathway in the *PFK1Δ* strain, leading to a decrease in glucose uptake and utilization, which subsequently decreased ethanol production efficiency. The cell wall contact tests (Supplemental Fig. S1) and supernatant replacement (Supplemental Fig. S3) demonstrated that the growth of *E. coli* was impaired due to the exclusion of cell wall contact and other substances in the culture medium. The growth of *E. coli* was also inhibited by different concentrations of ethanol (Supplemental Fig. S4), and the ethanol content on the side inoculated with *E. coli* was higher than that on the uninoculated blank group (*P* < 0.01) (Supplemental Fig. S2B). The acetic acid secreted by *E. coli*, along with other detrimental substances to yeast cell growth, permeates into the yeast-containing MIC bottle through the filter membrane inducing the yeast cells to respond to this specific stimulus. The ethanol produced by *S. cerevisiae* showed directional flow and consistently moved towards the side exposed to external stimuli. These results further suggest the potential of ethanol as a defense weapon for *S. cerevisiae*. As a result, the decline in ethanol production efficiency caused by the *PFK1* deletion diminishes the interspecies competitiveness of *S. cerevisiae*. The competitiveness of microorganisms can be evaluated by measuring their ability to grow in a competitive environment (Khonsari and Kollmann 2015). Therefore, the growth of *S. cerevisiae* and *E. coli* in mixed culture (Fig. 4 A and B) also suggests that *PFK1Δ* had the weakest interspecific competitiveness among the three strains in co-culture with *E. coli*.

**Fig. 5:**
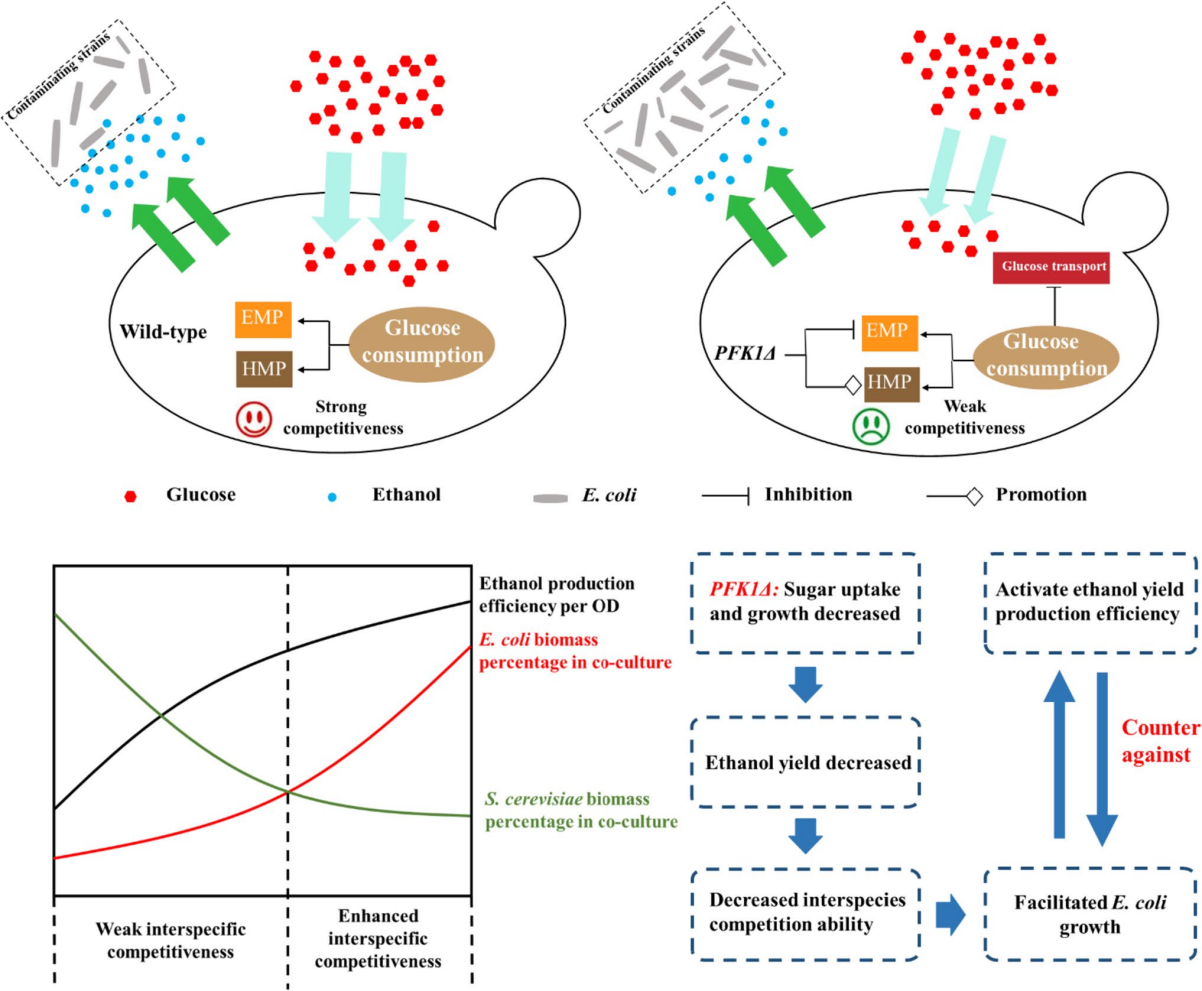
Overview of the *PFK1* gene deletion on the sugar uptake and utilization, ethanol production, and interspecific competitive ability of *Saccharomyces cerevisiae*

The expression of key genes in ethanol synthesis decreased in the early stage of the mixed culture (Fig. 4C), suggesting a lower ethanol production efficiency of *PFK1Δ* strain in the co-culture. The reduced ethanol yield resulted in the rapid growth of *E. coli* in the co-culture with the *PFK1Δ* strain. When microorganisms are subjected to more stress from environmental factors or other microorganisms, they exhibit certain defense behaviors (Guan et al. 2017); and with the extension of the time of feeling stress, the ability of microorganisms to respond to proactive or passive stress gradually increases. Accordingly, during the later stages of the mixed culture, the expression of genes critical to ethanol synthesis in the *PFK1Δ* strain increased (as shown in Fig. 4D), leading to an increase in ethanol synthesis, which in turn served to counteract the contaminating microorganisms. At this time (i.e., 16 h of co-culture), the biomass of *E. coli* in co-culture with the *PFK1Δ* strain reached the maximum, and the *PFK1Δ* strain enhanced its ability to produce ethanol as a defensive weapon to counter the enhanced threat from *E. coli*. However, in the early stage, the glucose uptake capacity and ethanol production capacity of *PFK1Δ* were weak; therefore, *PFK1Δ* was more susceptible to other microorganisms (i.e., *E. coli* here) than the wild-type strain and the other four knockout mutants (Fig. 5).

The deletion of *PFK1*, a crucial gene in the EMP pathway, can slow down the utilization of intracellular glucose and decrease the glucose uptake of yeast cells, leading to more residual glucose in the medium for the *PFK1Δ* strain to use for growth and reverse overshoot at the stationary stage. Meanwhile, the slower growth of the *PFK1Δ* strain in the early stage also reduced the yield of ethanol, which could be used as a defensive weapon to counter the competitiveness or thereat from *E. coli*, making the *PFK1Δ* strain less competitive than the wild-type strain while being co-cultured with other microorganisms (i.e., represented by *E. coli* here). The *PFK1Δ* strain’s decreased interspecies competitiveness led to increased biomass accumulation in the co-cultured *E. coli*, which in turn enhanced the *PFK1Δ* strain’s ethanol production efficiency and allowed it to counter the threat posed by the *E. coli* at the stationary stage. The results of this study could be valuable in understanding the regulation of the *PFK1* gene on the growth and interspecies microbial competition behavior of *S. cerevisiae*. Moreover, this finding that regulating the expression of the *PFK1* gene could enhance the growth ability, yield, and tolerance of *S. cerevisiae* has significant implications for the selection and optimization of strains in industrial fermentation. This could improve the efficiency and yield of the fermentation industry by adjusting the competitiveness of different yeast strains in the fermentation process. Additionally, regulating the expression level of *PFK1* gene could also control the competition between *S. cerevisiae* and other microorganisms in food processing, improving the fermentation efficiency and taste and nutritional value of products. In future studies, the introduction of a target gene plasmid with an inducible promoter can be employed to achieve expression of the *PFK1* gene over time periods. Such strategy could allow for rapid biomass accumulation and enhance interspecies competitiveness during the early stage, ultimately resulting in *S. cerevisiae* strains with heightened tolerance in the later stage. The findings of this study hold practical applications in various fields such as wine making, biofuel production, and other food or chemical production.

## Supplementary Information

Below is the link to the electronic supplementary material.

Supplementary file1 (PDF 683 KB)

## Data Availability

All data generated or analyzed during this study are included and available in this published article (and its supplementary information files).
