# Supplementary material for: Regulation of the PFK1 gene on the interspecies microbial competition behavior of Saccharomyces cerevisiae
Source: Appl Microbiol Biotechnol. 2024 Mar 22;108:272. doi: 10.1007/s00253-024-13091-9 (PMC10959778; doi:10.1007/s00253-024-13091-9)
Supplement: Supplementary file 1 [file 253_2024_13091_MOESM1_ESM.pdf]

Journal name: Applied Microbiology and Biotechnology

Manuscript Title: Regulation of the *PFK1* gene on the interspecies microbial competition behavior of *Saccharomyces cerevisiae*

The name(s) of the author(s): Caijuan Zheng, Shuxin Hou, Yu Zhou, Changyuan Yu, Hao Li

The affiliation(s) and address(es) of the author(s)

The affiliation and address of Caijuan Zheng, Shuxin Hou and Changyuan Yu: College of Life Science and Technology, Beijing University of Chemical Technology, Beijing 100029, People's Republic of China.

The affiliation and address of Yu Zhou and Hao Li: School of Public Health, Jining Medical University, Jining 272067, People's Republic of China.

The e-mail address, telephone and fax numbers of the corresponding author: E-mail address: lihaoh@163.com; lihao@mail.jnmc.edu.cn; Tel: +86- 0537-3616333; Fax: +86-0537-3616777.

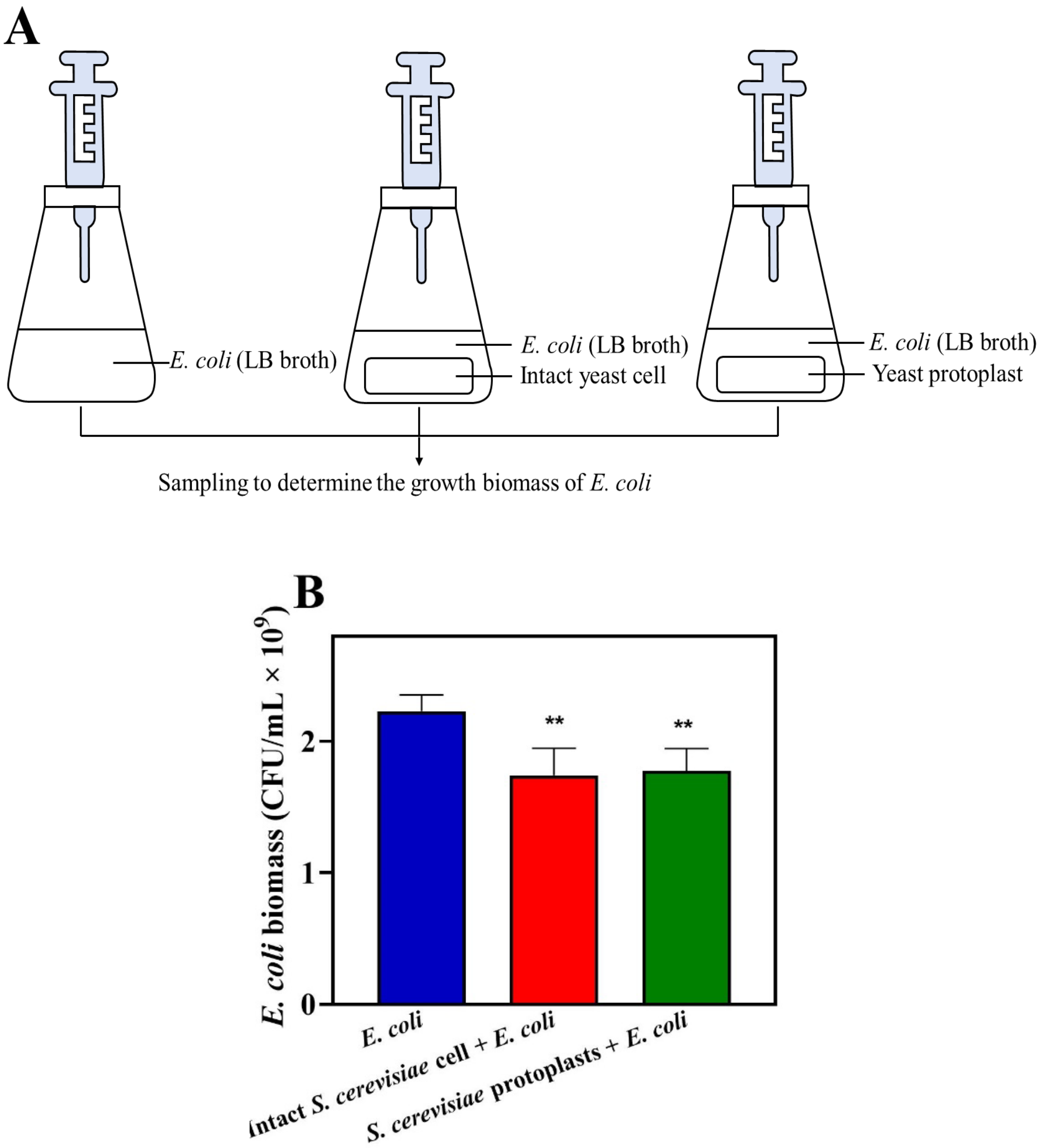

Fig. S1 Exclusion of inhibitory effect of *S. cerevisiae* cell wall contact on the growth of *E. coli*. (A) Experimental grouping design. Three conical bottles were inoculated with the same amount of *E. coli*, and intact *S. cerevisiae* cells and *S. cerevisiae* protoplasts were added to the experimental groups. Three groups of parallel samples were set up in the experimental groups and the control group. (B) After *E. coli* was cultured for 9 h, the intact *S. cerevisiae* cells and *S. cerevisiae* protoplasts were added in the experimental groups, and then cultured for 1 h. The biomass of *E. coli* was calculated by smearing the plate. ***P*<0.01 compared to the control group.

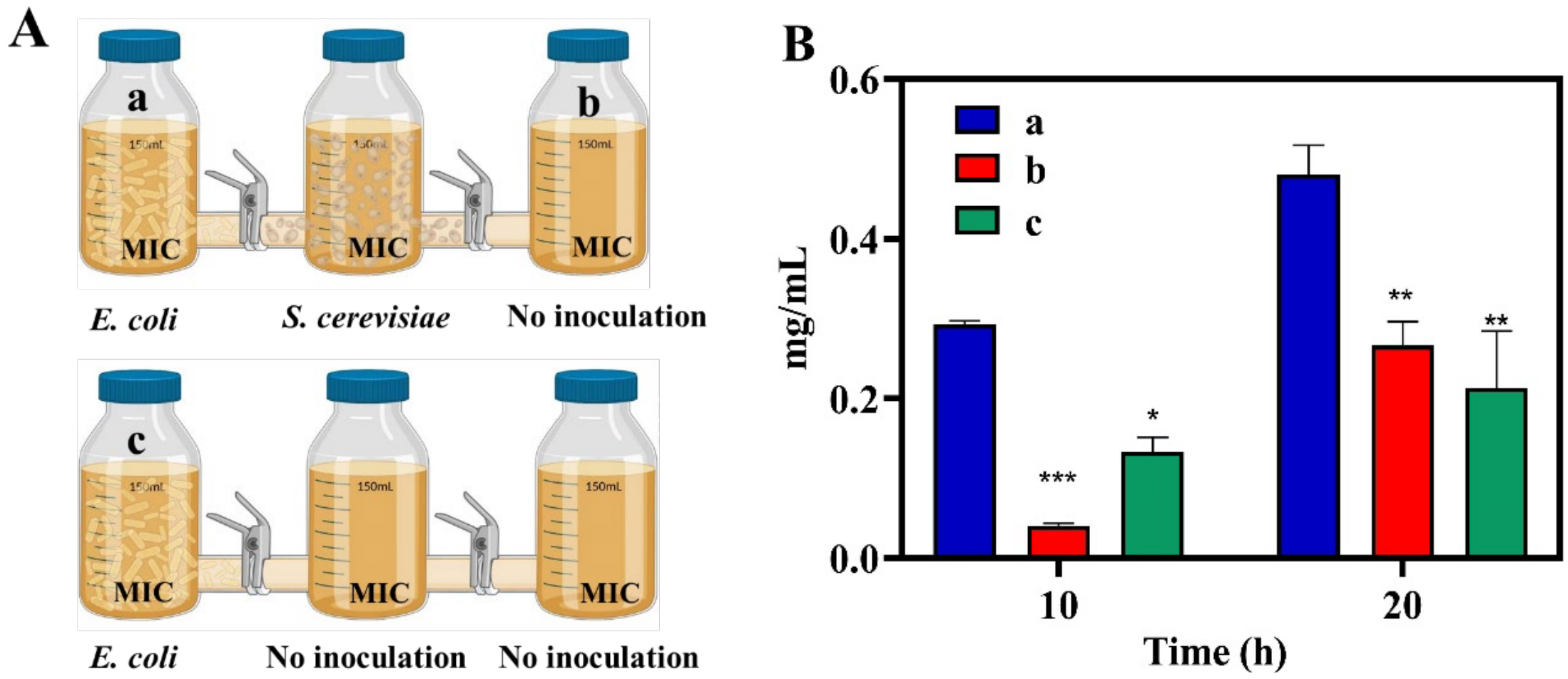

Fig. S2 Effect of *Escherichia coli* on ethanol fluidity in the MIC bottles. (A) Experimental grouping design. Three sets of parallel experiments for each set of devices was set up. (B) In the presence of *E. coli*, the concentration distribution of ethanol produced by *Saccharomyces cerevisiae* in the intermediate MIC bottle flows to both sides. The experimental group (a) was inoculated with *E. coli*, the blank group (b) was not inoculated with *E. coli*, and the control group (c) was cultured separately. **P*<0.05 compared to the experimental group (inoculated with *E. coli*). ***P*<0.01 compared to the experimental group (inoculated with *E. coli*).

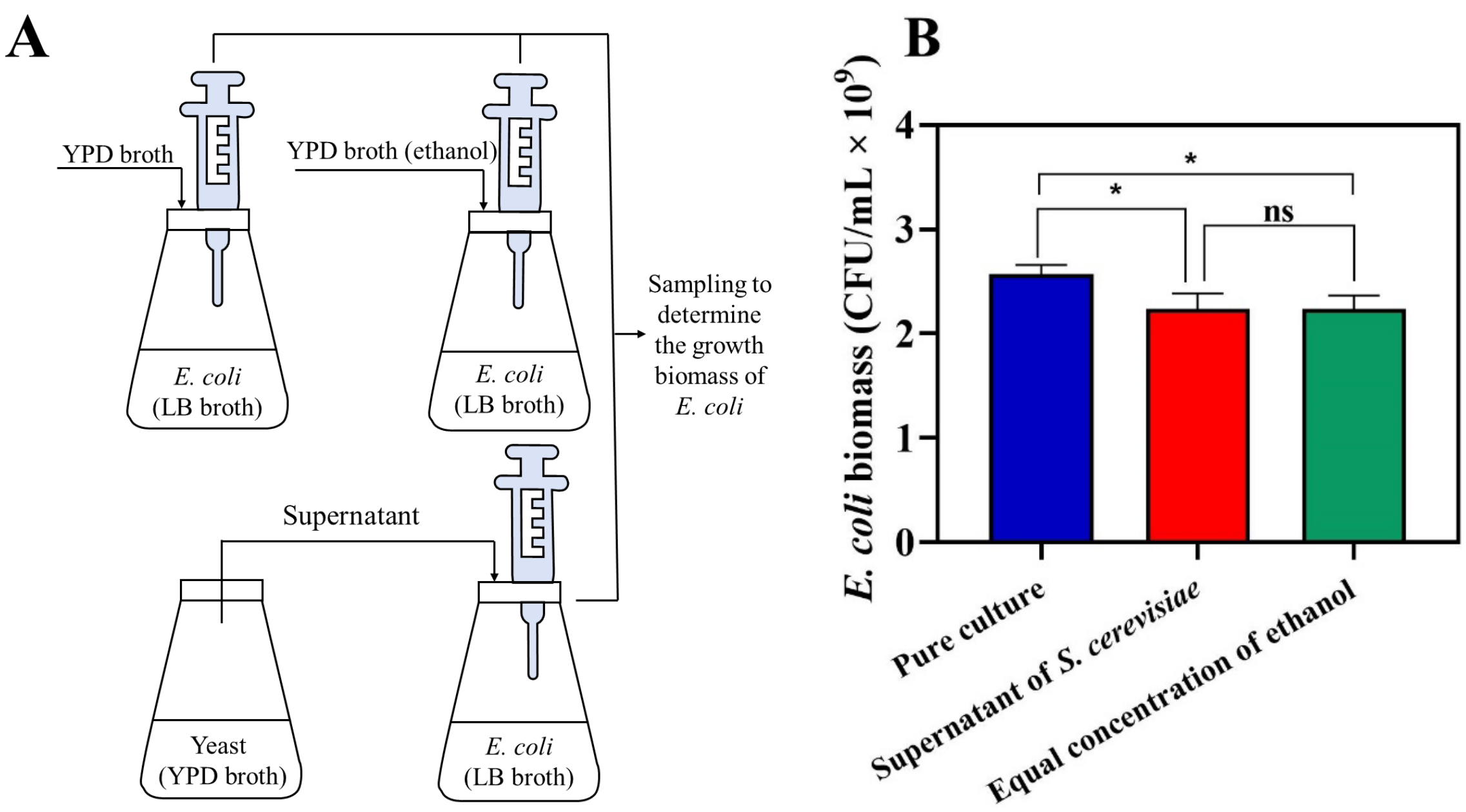

Fig. S3 Exclusion of inhibitory effect of other substances in the supernatant of *S. cerevisiae* on the growth of *E. coli.* (A) Experimental grouping design. The *E. coli* cells cultured for 10 h were added the same amount of YPD solution (control group), the supernatant of *S. cerevisiae* cultured for 10 h (experimental group) and YPD solution containing ethanol (the concentration of ethanol equal to the concentration of ethanol produced by *S. cerevisiae* for 10 h) (experimental group). (B) The biomass of *E. coli* in the control group and experimental groups was calculated by plate counting method after 1 h culture. *$P<0.05$ compared to the control group (pure cultured *E. coli* without supernatant replacement).

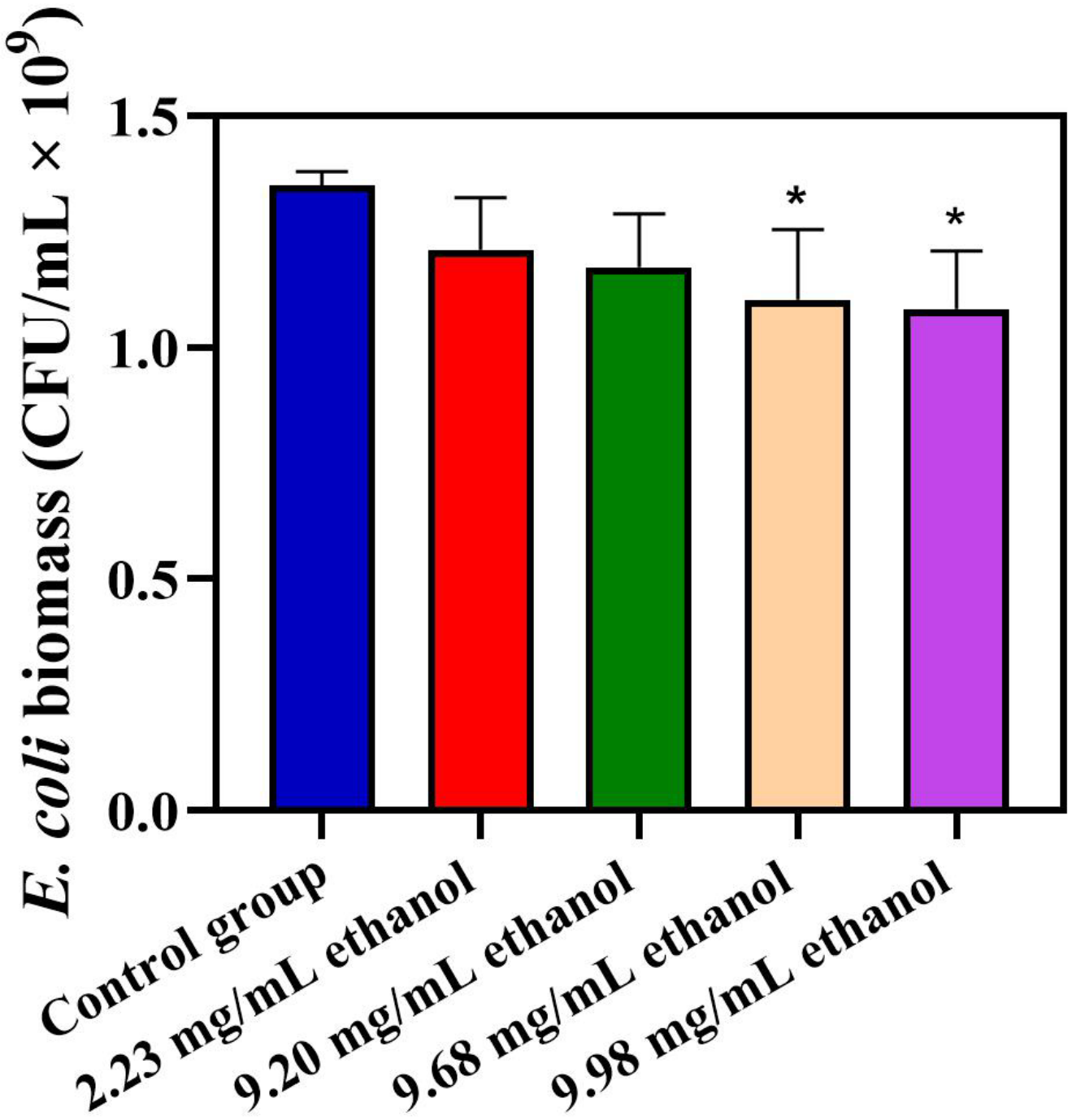

Fig. S4 Effects of different concentrations of ethanol treatment on the growth of *E. coli.* In the experimental groups, different concentrations of ethanol were added respectively, and the ethanol concentration was set to the ethanol concentration produced by *S. cerevisiae* at 10h, 14h, 16h and 20h, respectively. $^{*}P<0.05$ compared to the control group (no ethanol added).
